# Insights for inclusive upstream engagement with migrant farmworkers in agriculture: A case study from the US–Mexico border

**DOI:** 10.1007/s13280-026-02383-1

**Published:** 2026-04-04

**Authors:** Sohinee Bera, Cristian Salazar De Leon, Bruce V. Lewenstein

**Affiliations:** 1 Department of Communication, Cornell University, Ithaca, NY USA; 2 School of Plant Sciences, University of Arizona, Tucson, AZ USA

**Keywords:** Inclusion, Migrant farmworkers, Plant biotechnology, Stakeholders, Upstream engagement

## Abstract

Amid climate change, plant biotechnologies are being developed to enhance the efficiency and sustainability of agricultural systems. However, their social and ethical implications remain underexplored through upstream engagement, which refers to the early engagement and involvement of stakeholders to shape research decisions. Using a case study from the U.S.–Mexico border, we reflect on the challenges and opportunities from conducting upstream engagement with migrant farmworkers. Tensions in language translation, issue framing, literacy, and tacit knowledge are particularly amplified when engaging with these kinds of communities, potentially shaping how participants interpret scientific information and express their views. Most participants reported concern that using gene editing on plants could increase social inequality, reinforcing the ethical imperative to engage overlooked stakeholders in agricultural research. We suggest that upstream engagement must be carefully designed to fit the lived realities of marginalized stakeholders to ensure their perspectives can meaningfully inform innovation.

**Supplementary Information:**

The online version contains supplementary material available at https://doi.org/10.1007/s13280-026-02383-1.

## Introduction

In the face of climate change, pressures to use fewer resources in agriculture are driving the next green revolution toward technocentric, digital solutions (Rose and Chilvers 2018). Emerging plant biotechnologies are often hailed as a “silver bullet” for enhancing agricultural efficiency and productivity (Barrett and Rose 2022, p. 163). However, the unintended social and ethical consequences of these biotechnologies remain underexplored, especially their impacts on socially marginalized communities in agriculture.

Agricultural and food systems are tied to human and environmental health in intricate ways, beyond food security or sovereignty (Ajates et al. 2025). As such, emerging agricultural biotechnologies can have multidimensional impacts and consequences on people, plants, and the environment. Ayris et al. (2023) emphasize that plant biotechnologies can raise challenges for data ownership and privacy, traditional agricultural practices, labor issues, and more. Several scholars have called for a more responsible approach to the technocentric transformations in agriculture, urging more *upstream engagement* (Rose and Chilvers 2018; Bronson 2019; Ayris et al. 2023; McGrath et al 2023)*.* This kind of engagement refers to activities that are conducted early in the development of novel areas of scientific work to understand contextual issues that publics are considering (Wilsdon and Willis 2004).

Emerging agricultural technologies are likely to affect migrant farmworkers’ labor, livelihoods, and exposure to risks, as they are intertwined with agricultural systems. Farmworkers report that these innovations can increase stress, intensify fear over disciplinary action, and heighten workplace competition (Doggett et al. 2024). Including farmworkers’ perspectives is essential to ensuring procedural justice and democratic ideals of scientific participation (Jasanoff 2003). In addition, upstream engagement can help anticipate broader social justice concerns, as farmworkers’ experiences with agricultural technology may shape how others, such as consumers, policymakers, or scientists, perceive the fairness and impacts of these innovations. However, the practice of upstream engagement is limited in the USA (Pidgeon 2020), particularly in its application with marginalized communities. In the USA, for example, migrant farmworkers are among the most vulnerable communities and rarely are their values and visions considered in the global project of agricultural biotechnology (McGrath et al 2023).

This perspective article reflects on inclusive practices for upstream engagement with the U.S. migrant farmworkers, drawing on a case study conducted at the US–Mexico border. We first review the literature on upstream engagement and migrant farmworkers’ experiences in agriculture. We then describe and reflect on our engagement activity to explore how issues of upstream engagement become amplified when working with marginalized communities. Building on this, we identify insights and strategies for designing and conducting the engagement activity. Throughout, our reflections offer practical guidance for researchers, policymakers, and practitioners interested in sustainable agriculture, inclusive engagement, and responsible research and innovation.

## Considerations for effective upstream public engagement

Upstream engagement refers to public engagement activities conducted early in the development of novel areas of scientific and technological work, where publics significantly consider the technology itself and any potential associated social and ethical issues (Wildson and Willis 2004; Pidgeon 2020; Rodgers-Hayden 2023). Compared to downstream approaches, where key decisions about a technology, risk, or research have already been made and engagement often focuses on educating or convincing stakeholders, upstream engagement involves stakeholders early on so that their values, visions, and concerns can shape development and outcomes (Pidgeon 2020). Engagement activities can range in level of involvement and mechanisms, from open house meetings and public opinion surveys to citizen advisory committees and public hearings (Rowe and Frewer 2005).

Drawing from normative, substantive, and instrumental arguments, scholars suggest that engaging publics upstream upholds democratic ideals of scientific participation (Abelson et al 2003; Stilgoe et al. 2014), fosters legitimacy and trust in institutions (Rowe and Frewer 2000; Abelson et al 2003), generates higher quality scientific and technological outcomes and developments (Pidgeon 2020), and ensures that innovations align with stakeholder values and needs (McGrath et al 2023). Moving upstream can bring additional benefits beyond traditional forms of public engagement, such as eliciting public imaginaries of what kinds of trajectories a technology might take, understanding desired and undesired consequences, and helping mitigate public controversy (Pidgeon 2020).

Simply involving stakeholders in upstream engagement does not guarantee more effective, efficient, or responsive science outcomes (Haywood and Besley 2014). Certain kinds of public deliberation activities in food and agricultural research have been criticized as being performative or manipulative, as they may give the illusion of participation without providing legitimate agency to stakeholders (Ajates et al. 2025). Rather, these kinds of activities enable organizers to persuade and convince stakeholders of their agenda, leading to the status quo persisting (Arnstein 1969).

Responsible upstream engagement depends not only on institutional reflexivity, but also of those organizing and mediating engagement (Chilvers and Kearnes 2019). This perspective shifts beyond inclusion, representation, and participant learning, to reflecting on how participation itself works. In other words, it critically examines what counts as the issue, who counts as the participant, and what kinds of futures, consequences, and norms are reinforced through engagement practices (Chilvers and Kearnes 2019). Positioning engagement as a reflexive practice in this way sets the critical perspective that guides this paper.

Providing balanced information in upstream engagement is challenging, as the topics subject to upstream engagement generally have little a priori public familiarity (Pidgeon 2020). Detailed, unbalanced information can bias participants toward the alignment of the institution, while overly broad information may make it difficult to gather public responses that are specific enough to guide development (Bruce and Bruce 2019).

Time and resource constraints are another major consideration (Lantz et al. 2001; Littlefield et al. 2024). Meaningful engagement requires open, sustained deliberative spaces, so participants have enough time for significant consideration (Pidgeon 2020). However, for marginalized communities, participating in such activities can take away time, resources, and capacity from addressing other competing issues (George et al 2014; Viswanath et al 2022). For example, migrant farmworkers face structural issues such as unsafe living and working conditions, difficulty accessing health care and worker’s services, and legal vulnerabilities (Haley et al 2020). One way to alleviate some of the potential burden of engagement is by ensuring reciprocity. Yet, while stakeholders may seek immediate information or benefits from their participation, the long timelines of upstream research mean that tangible benefits may not be available for years (Bamzai-Dodson et al 2021). Lastly, deciding *who* counts as a stakeholder remains a persistent challenge across both upstream and traditional engagement approaches (Abelson et al. 2003; Rowe and Frewer 2004; Pidgeon 2020).

## Migrant farmworkers’ experiences in agriculture

Within digital agriculture, the social science literature largely focuses on farmers and their motivations for adopting technologies (McGrath et al 2023), leading to public engagement only being conducted when the desired outcomes of an agricultural project were not achieved. The goal of technology adoption then frames ethical and social issues as barriers to be overcome (Ayris et al. 2024), rather than using engagement to develop a responsive, two-way research dialogue between publics and scientists. Furthermore, the participation of marginalized stakeholders, such as migrant farmworkers, is limited in public engagement activities in agriculture (McGrath et al. 2023). Migrant farmworkers are rarely considered as relevant stakeholders in the development of agricultural technologies, largely because they tend to work with hand-harvested crops that have not traditionally been the focus of biotechnology research. With the ongoing development of emerging plant biotechnologies on a variety of crops, it is crucial to understand farmworkers’ perspectives as these innovations take shape.

In Mexico, for example, economic hardship often drives citizens to cross the border daily for better-paying work in the USA, leading them to tolerate working conditions that, while poor by national standards, are relatively better than those in Mexico (Rosales et al. 2012). Although some workers cross the border legally, cross-border politics heightens farmworkers' risks related to their legal status, labor mobility, and access to social services and health care, rendering them as what Cohen (2019) calls “essentially bonded laborers” (p. 137).

Research conducted on migrant farmworkers in Germany reveals that emerging agricultural technologies can facilitate digitally enabled surveillance, introduce new performance metrics, lead to wage theft, and ultimately undermine the autonomy of workers, as they feel pressured and controlled by these technologies to maximize efficiency at the expense of their own well-being (Prause 2021). Similarly in Canada, agricultural technologies that track labor cause migrant farmworkers to fear surveillance and consequent disciplinary action, stress over not meeting expectations, and feel competitive against other farmworkers from performance tracking (Doggett et al 2024). Other farm laborers in the USA have expressed concern that automated agricultural technologies will replace them and their labor (Carolan 2020), disrupting their legal immigration statuses.

## Motivation for upstream engagement with migrant farmworkers

Our interest in developing upstream public engagement activities emerged from the development of a publicly funded and multi-institutional agricultural research center: Center for Research on Programmable Plant Systems (CROPPS). CROPPS is a transdisciplinary research center using tools from synthetic and molecular biology, bioengineering, plant breeding and quantitative genetics, agronomy, horticulture, robotics, artificial intelligence, computer modeling, and social sciences to develop *programmable plants* (CROPPS, n.d.). CROPPS defines programmable plants as plants and technologies with autonomous capabilities that can sense environmental factors and respond, independently gather and integrate data, and transmit this information to people in an accessible format. Current examples of programmable plants in development include plants engineered to act as *living sensors*, such as those that change color when experiencing nitrogen stress (Collegiate Inventors Competition, n.d.), or receive signals to close their stomata to improve water and nitrogen use efficiency. These engineered plants are then scaled and coordinated to form part of an emerging *internet of living things,* which include biohybrid robots that can sense soil chemistry and autonomously decide when to add more fertilizer (Mishra et al. 2024) and cloud-based AI tools for analytics and decision support.

Since CROPPS’ inception, public engagement has been an integral part of the center’s work to understand public visions and integrate this into research design and practice. Insights from public engagement activities are continually shared with scientists at the center, providing opportunities for them to consider stakeholder perspectives as they develop these technologies. The opportunity to survey and engage with migrant farmworkers was of interest to scientists at CROPPS, during the second year of a projected 10-year long project, as technologies were being prototyped.

## Día del Campesino: Day of the farmworker

Yuma County, Arizona, known colloquially as the United States’ “lettuce capital”, is responsible for 90% of all leafy vegetables grown in the nation during the winter months (Whitney 2022). About 40,000 farmworkers are estimated to work in Yuma County alone, including Mexican citizens commuting legally, daily across the border (Rangel 2023). Migrant workers are essential to the farming community and vegetable industry, as most leafy vegetables must be harvested, cleaned, and packed by hand.

We conducted a public engagement activity with a survey during “*Día del Campesino*” (Day of the Farmworker) in December 2023. This is a one-night annual farmworkers event in Yuma County located a few feet away from the international border of the USA and Mexico. The event, organized by a local farmworker community organization, brings together over 3000 migrant farmworkers and their families to provide free health services, immigration clinics, food, and cultural activities. The event is held from 2 to 6 AM to accommodate the majority of farmworkers in the region, who live in Mexico and are traveling across the border before beginning their workday. At the event, we hosted an outreach booth, where we invited migrant farmworkers to take a survey in Spanish on their perceptions and attitudes toward plant biotechnology, scientists, and public engagement activities. We received 59 completed surveys, with 54 completed on paper and five delivered orally.

In the following sections, rather than focusing solely on the survey data, we present our reflections and insights from the experience, which were documented through observational notes on participant interactions, questions, and event details taken in real time or immediately after the event. The notes and reflections from the experience were later reviewed collaboratively among all authors to identify recurring patterns and key lessons.

## Critical reflections on engagement

### Recruitment

Our engagement with this community of migrant farmworkers was facilitated by one of the author’s personal connections and shared cultural and regional background with migrant farmworkers in this area. These connections, together with collaboration with a local farmworker community organization that allowed us to be at the event, could have helped enhance the authenticity and cultural relevance of the engagement (Doggett et al. 2024). At the same time, we recognize that even with the shared background and organizational support, inherent power differentials between academic researchers and farmworkers might have influenced how participants engaged with us. For instance, when asked about trust in information sources on agricultural topics, 99% of participants reported that they had a fair to great deal of trust in university scientists, compared to 89% in industry scientists, and 93% in family and friends. While these numbers from the survey suggest a high trust in academic sources, they may rather reflect social desirability bias as participants might have felt compelled to answer in a particular way when interacting with us as academics.

Based on feedback from the farmworker community organization, we selected items such as CROPPS-branded lunchboxes, T-shirts, water bottles, and notebooks as compensation, given their practicality for workers’ everyday use. Organizers advised that recruitment would be challenging at this event, where participants were more likely to prioritize food, legal and health services, and time with their community over talking to researchers. To initially encourage participation, we placed a variety of compensation items on the table at the outreach booth and asked passersby if they were interested in taking a survey on programmable plants. While this drew substantial interest, the visibility of the compensation items contributed to a high volume of visitors in a short period of time, making it difficult to sustain in-depth conversations and conduct surveys in a consistent manner.

In response, we adjusted our approach by placing chairs in front of the booth and displayed only two or three compensation items at a time, rather than the full set. We shifted our oral recruitment to inviting participants into conversation and asking whether they were interested in learning more about programmable plants. With this shift, we noticed how visible compensation and exhibit design did not simply motivate participation, but actively configured how engagement unfolds, shaping the pace, form, and depth of interaction (Chilvers and Kearnes 2019). Our oral recruitment adjustment could have increased participant’s autonomy to opt into these conversations, rather than creating an implicit expectation to complete the survey in exchange for compensation.

### Survey design and instrument

The survey questionnaire used during the engagement was reviewed, edited, and approved by the community organization to ensure it would be appropriate for migrant farmworkers. Based on their guidance, surveys were designed to be monolingual in Spanish, rather than having both English and Spanish on one survey, to reduce text and potential confusion. The organization also recommended that questions be close-ended and simplified, minimizing the time and effort required to participate. Accordingly, the final questionnaire (see Supplementary Material) was close-ended, despite the well-known limitations in close-ended questionnaires constraining participants’ ability to offer insights beyond predefined questions or draw connections to other issues (Stoneman et al. 2013). The survey took approximately 10 min to complete.

While the challenges of providing balanced information in upstream engagement are known (Bruce and Bruce 2019; Pidgeon 2020), this experience revealed that issues of language translation, issue framing, and literacy are amplified when engaging with marginalized communities. Our survey revealed that participants primarily spoke Spanish and roughly half had not completed schooling beyond middle school education (see Supplementary Material). As Lewenstein (2022) notes, limited access to scientific resources often leaves marginalized groups less able to benefit from the reliable knowledge science produces. This added an additional layer of difficulty when trying to provide balanced information on a technical concept in an accessible format.

During the event, other community organizers suggested that the text prompts defining our technical terms might have been too lengthy for those with literacy and educational challenges, even after being translating into Spanish. For example, the definition of “plant bioengineering” read: “*Plant bioengineering uses technology to improve our scientific understanding of plants so that people can do things like grow plants more efficiently and help crops survive in changing climate conditions.”* Given participants likely limited prior familiarity with these terms, the framing of the provided definitions likely shaped their responses. When presented with the plant bioengineering definition, 81% reported that this technology provides far to some greater benefits to society, and 96% reported that they support the use of plant bioengineering to improve agricultural productivity.

The community organization also recommended simplifying text, incorporating visual graphics into question and response items, such as icons of happy or sad faces to indicate agreement levels, while simultaneously orally delivering questions. This recommendation aligns with research that suggests picture-text congruency can aid viewers’ learning, retention, and recall of information (Graber 1996; Eitel et al. 2013).

During survey administration, several respondents mentioned that they felt there were too many response options to choose from, expressing discomfort for the standard Likert-style 5-point scale. They felt that they either agreed or disagreed with statements and did not feel like there was a difference between “strongly agree” and “somewhat agree”. Other respondents felt restricted by not being able to select more than one response option, where they had to exclusively rank plant biotechnology goals. They felt that all the goals were equally important and were unsure how to rank them.

### Time and capacity

Across community-based research, time consistently emerges as a significant challenge (Lantz et al. 2001; Littlefield et al. 2024). In our case, this meant accommodating farmworkers’ schedules and engaging from the hours of 2–6 AM, which were most convenient for them. Beyond scheduling, migrant farmworkers at the event could have had more pressing social, health, and legal issues to attend to through the on-site clinics, constraining their capacity for engagement with researchers. These constraints might help explain the resulting sample size of 59 participants in our survey and reflect the broader challenges of engaging “hardly reached” communities (Viswanath et al. 2022).

Similarly, it takes time to witness any benefits that upstream engagement may produce (Bamzai-Dodson et al. 2021). In our case, we informed participants that their inputs would be shared with scientists at CROPPS, which has occurred on multiple occasions. Still in the early development stages, we cannot evaluate the influence of this or other upstream engagement activities on research design and practice, yet. Following a center-wide presentation and Q&A session with scientists at CROPPS on our engagement with migrant farmworkers, we distributed a follow-up survey to the presentation attendees. Survey responses from the scientists included expressions of “appreciation for the opinions of a group we rarely hear from as scientists” and “glad that this kind of work is being conducted as the opinions of a lot of migrant workers are usually disregarded”.

In our efforts to continue research with other regional farmworker organizations, we were informed that this project did not align with the priorities of those organizations. These organizations were largely focused on the legal, labor, and human rights concerns of migrant farmworkers. While we attempted to position this project as supportive of, rather than competing with, these organizations’ goals, the reality is that there may be more urgent issues to address for marginalized stakeholders.

## Discussion

Central to our reflection is a guiding question: What does it take to responsibly engage migrant farmworkers about emerging agricultural technologies, and what does such engagement reveal about power, inclusion, and the conditions under which their perspectives can meaningfully inform research?

Our experience highlights several key conditions for meaningful engagement. Engagement requires flexible timing and accessible, culturally, and linguistically appropriate materials. For survey-based engagements, local language instruments, simplified text, reduced response options, and visual-verbal-written redundancy may help mitigate literacy barriers. We also recognized the importance of tacit knowledge in understanding *how* to take a survey. Through conversations, several farmworkers indicated that they were surprised we wanted to know their opinions and that they had little to no experience with surveys of this kind. This lack of familiarity could have influenced their comfort during recruitment and their responses, as participants had to navigate exercising agency in deciding to participate, interpreting technical definitions, and answering within normative survey formats, such as Likert-style questions. Such considerations would likely differ when recruiting other social groups, such as American farmers or consumers, whose motivations, expectations, capacities, and prior experiences with surveys vary widely.

Despite our efforts to craft balanced and neutral messaging and keep language consistent with our previous surveys, careful wording and framing may lead to more reflective risk perceptions. Bellamy and Lezaun (2017) discuss the idea of ‘unframing’ as a methodological principle of public engagement, to turn scientific topics into objects of deliberation without necessarily implying a commitment to their reality. How to “unframe” topics in upstream engagement activities with marginalized communities remains an open question. For example, engaging farmworkers to provide input on agricultural biotechnologies that are actively being developed might be considered “too late”, as there was not an earlier opportunity for farmworkers to consider whether this kind of technological innovation is necessary at all.

Inclusive engagement also requires careful consideration of participants’ broader priorities and competing demands, as time spent on engagement can take away time spent on addressing other issues. In this case, farmworkers’ capacity to engage may have been limited by their intersecting layers of marginalization, revealing the broader power asymmetries in who is able to participate in shaping technological decisions and under what conditions. Such constraints illustrate that the typical recommended upstream engagement formats, such as multi-session deliberative workshops held over an extended period of time (Pidgeon 2020), are not always feasible for marginalized communities without careful planning and consideration.

For future work, integrating trusted community organizations, providing tangible and immediate reciprocity, and establishing sustained engagements are critical so that farmworkers’ perspectives can influence research over time. Our goal of upstream engagement was to create a pathway for farmworkers to influence how these technologies are developed, ideally ensuring that the technologies support and align with their needs. However, the very conditions that limit their voice and make this kind of engagement necessary also constrain their ability to participate. In this way, upstream engagement is both necessary to address these structural inequalities but challenging to conduct meaningfully because of these inequalities.

Nonetheless, the survey findings illustrate why such engagement is critical. When asked about how using gene editing on plants might affect social inequality, 76% of respondents indicated that it would either strongly or somewhat increase social inequality. These responses reveal that migrant farmworkers are aware of and concerned about the potential social impacts of emerging technologies, emphasizing the ethical importance of incorporating their perspectives into research.

Reflecting on *Día del Campesino,* the case illustrates both the promises and tensions of upstream engagement. The engagement activity did open up pathways for dialogue, bring to light some ethical concerns, and at least temporarily increase scientists’ awareness of migrant farmworkers’ perspectives. At the same time, without addressing structural barriers and providing meaningful mechanisms of engagement that work within the lived realities of farmworkers, upstream engagement could risk reproducing tokenistic forms of participation. In this way, our experiences add to the growing body of work on upstream public engagement (Wildson and Willis 2004; Rogers-Hayden and Pidgeon 2007; Pidgeon et al 2011; Pidgeon 2020; Rogers-Hayden 2023), while advancing a key literature gap on understanding inclusive practices with socially marginalized communities.

## Conclusion

Our exploration into migrant farmworkers in Yuma County, Arizona, revealed complexities of upstream public engagement with marginalized communities in the context of plant biotechnologies. We gained insights into how to design and deliver upstream activities to better serve marginalized stakeholders. Carefully balancing marginalized stakeholders’ limited time and capacity, providing balanced and accessible information, and understanding power asymmetries between researchers and participants will remain ongoing challenges for the practice of upstream engagement. In addition, we learned that translating our materials is likely not enough for a Spanish-speaking community with limited formal education and literacy barriers. Transforming information into alternative formats with visual, verbal, and written congruence may improve comprehension and understanding. Ultimately, as agricultural innovations continue to evolve in response to the changing climate, it is increasingly important to develop effective and inclusive upstream approaches to understand and mitigate the unintended social consequences of these innovations.

## Supplementary Information

Below is the link to the electronic supplementary material.

Supplementary file1 (PDF 156 KB)
