# Supplementary material for: Insights for inclusive upstream engagement with migrant farmworkers in agriculture: A case study from the US–Mexico border
Source: Ambio. 2026 Apr 4;55(11):2633–40. doi: 10.1007/s13280-026-02383-1 (PMC13661884; doi:10.1007/s13280-026-02383-1)
Supplement: Supplementary file 1 [file 13280_2026_2383_MOESM1_ESM.pdf]

Insights for Inclusive Upstream Engagement with Migrant Farmworkers in Agriculture: A case study from the US-Mexico border

For submission at *Ambio: A Journal of Environment and Society*

Supplementary material: Survey Questionnaire & Participant Demographic Data

The follow pages includes the survey questionnaire text in English and Spanish (pages 1- 9) and the survey participants demographic data (page 10). The following pages do not include author details.

Dia Del Campesino Questionnaire

1. How much trust do you have in information about agricultural topics from university scientists? *(Please check one of the following answers).*

Cuánta confianza tiene en la información sobre temas de agricultura procedente de científicos universitarios? (*Por favor, marque una de las siguientes respuestas*).

A great deal/ **Mucha confianza**

A fair amount/ **Algo de confianza**

Not very much/ **No mucha confianza**

None at all/ **Nada confianza**

2. How much trust do you have in information about agricultural topics from private industry/company scientists? *(Please check one of the following answers).*

Cuánta confianza tiene en la información sobre temas de agricultura procedente de científicos de la industria privada/de compañías? (*Por favor, marque una de las siguientes respuestas*).

A great deal

A fair amount

Not very much

None at all

3. How much trust do you have in information about agricultural topics from family and close friends? *(Please check one of the following answers).*

Cuánta confianza tiene en la información sobre temas de agricultura procedente de su familia y amigos? (*Por favor, marque una de las siguientes respuestas*).

A great deal

A fair amount

Not very much

None at all

We would like to ask about your attitudes towards plant bioengineering. Plant bioengineering uses technology to improve our scientific understanding of plants so that people can do things like grow plants more efficiently and help crops survive in changing climate conditions.

Nos gustaría preguntarle sobre sus opiniónes sobre bioingeniería de plantas. La biotecnología en plantas utiliza tecnología para mejorar nuestra comprensión científica de las plantas, para que podemos cultivar las plantas más eficientemente y ayudar las cosechas sobrevivir en condiciones climáticas cambiantes.

4. In your opinion, does technology that helps us grow plants more efficiently create greater benefits or greater risks to society? (Please check one of the following answers).

¿En su opinión, la tecnología que nos ayuda a cultivar plantas de manera más eficiente crea mayores beneficios o mayores riesgos para la sociedad?

Far greater benefits/ **Muchos más beneficios**

Some greater benefits/ **Algo de beneficios**

Both equal risks and benefits/ **Beneficios y riesgos iguales**

Some greater risks/ **Algo de riesgos**

Far greater risks/ **Muchos mas riesgos**

5. There are many goals plant bioengineering technology is aiming to achieve in order to improve modern agriculture. Please rank the following goals from the most important (1) to least important (7).

Hay muchos objetivos que tecnología de biotecnología en plantas quiere lograr para que mejorar agricultura moderna. Por favor, clasifique los siguientes objetivos del más importante (1) al menos importante (7).

___ Reduce the amount of fertilizer that is needed to grow plants./ **Reducir la cantidad de fertilizante que se necesita para cultivar plantas.**

___ Reduce the amount of herbicide needed to grow plants./ **Reducir la cantidad de herbicida que se necesita para cultivar plantas.**

___ Reduce the amount of water that is needed to grow plants./ **Reducir la cantidad de agua que se necesita para cultivar plantas.**

___ Reduce the amount of pesticide that is needed to grow plants./ **Reducir la cantidad de pesticida que se necesita para cultivar plantas.**

___ Reduce carbon emissions in the agricultural industry./ **Reducir la cantidad de emisiones de carbono en la industria agricultura.**

___ Improve plant resilience to plant diseases and plant pests./ **Mejorar la resiliencia de las plantas a las enfermedades y pestes.**

___ Improve plant resilience to climate disasters (including climate change)/ **Mejorar la resiliencia de las plantas a los desastres climáticos (incluyendo cambio climático).**

6. Would you say you oppose or support the use of plant bioengineering in order to improve agricultural productivity? *(Please check one of the following answers).*

¿Cree que se opone o apoya el uso de la biotecnología en plantas para mejorar la productividad agricultura? (*Por favor, marque una de las siguientes respuestas*).

Strongly support/ **Apoya fuertemente**

Somewhat support/ **Algo de apoya**

Neither support nor oppose/ **No apoya ni opone**

Somewhat oppose/ **Algo de opone**

Strongly oppose/ **Opone fuertemente**

7. Do you think that improving agricultural productivity through plant bioengineering will have a positive impact on your community/family? *(Please check one of the following answers).*

Cree que mejorar la productividad de agricultura a través de la biotecnología en plantas tendrá un impacto positivo en su comunidad/familia? (*Por favor, marque una de las siguientes respuestas*).

Strongly agree/ **Acuerdo**

Somewhat agree/ **Algo de acuerdo**

Neither agree nor disagree/ **No acuerdo ni desacuerdo**

Somewhat disagree/ **Algo de disacuerdo**

Strongly disagree/ **Disacuerdo**

We would now like to ask about your attitudes towards gene editing and social inequality. Gene editing is a method for making specific changes to the DNA of a cell or an organism. Social inequality refers to when certain groups of people receive unequal opportunities and rewards compared to other groups.

Nos gustaría preguntarle sobre su opinión sobre la edición genética y la desigualdad social. La edición de genes es una forma de cambiar específicos en el ADN de una célula u

organismo. La desigualdad social se refiere a cuando ciertos grupos de gente reciben oportunidades y recompensas desiguales en comparación con otros grupos.

8. Do you think that using gene editing on plants will decrease or increase social inequality? (*Please check one of the following answers).*

Cree que el uso de la edición genética en plantas disminuirá o aumentará la desigualdad social?

Strongly increase inequality/ **Aumentar fuertemente la desigualdad**

Somewhat increase inequality/ **Aumentar un poco la desigualdad**

Neither increase nor decrease inequality/ **No disiminuir ni aumentar**

Somewhat decrease inequality/ **Disiminuir un poco la desigualdad**

Strongly decrease inequality/ **Disminuir fuertemente la desigualdad**

9. Do you feel comfortable harvesting/working with plants that have been modified through plant bioengineering or gene editing? *(Please check one of the following answers).*

Se siente cómodo cosechando/trabajando con plantas que han sido modificadas mediante biotecnología en plantas o con edición genética?

Extremely comfortable/ **Muy cómodo**

Somewhat comfortable/ **Algo de cómodo**

Not too comfortable/ **Incómodo**

Not at all comfortable/ **Muy incómodo**

Next, we would like to ask about your attitudes towards public engagement activities in science. Public Engagement with Science involves conversations between scientists and members of the public where both groups learn from each other about developments in science and their applications to society. This includes: the problems that communities view as worth solving; the information society needs and wants from scientists; the potential risks, benefits, and consequences of new technologies; building and sustaining trust among stakeholders; and finding common ground and working towards shared decisions about science-related controversies.

Nos gustaría preguntarle sobre sus opiniones sobre las actividades de participación pública en la ciencia. La intervención pública con la ciencia significa conversaciones entre

científicos y miembros del público para que ambos grupos aprenden sobre los avances de la ciencia y las aplicaciones en la sociedad. Esto incluye: los problemas que las comunidades consideran que importante para resolver; la información que la sociedad necesita y quiere de los científicos; los riesgos, beneficios y consecuencias potenciales de las nuevas tecnologías; creer y mantener la confianza entre las partes interesadas; y encontrar puntos en común y trabajar para tomar decisiones compartidas sobre controversias relacionadas con la ciencia.

Examples of public engagement activities in the role of a scientist, researcher, or other science communicator include giving a public talk or doing a demonstration for people who are not scientists. Doing an interview with a journalist or other media professional for a science news story or documentary is another example of public engagement. Public engagement can also include participating in town hall sessions where scientists and members of the public meet together to have conversations to learn about or find solutions for science-related issues. Online communication about science with people who are not scientists through blogs, websites, or social networks (e.g., Facebook, Twitter/X) also counts as public engagement.

Ejemplos de actividades de participación pública en la posición de un científico, investigador u otro comunicador científico incluyen: Una charla pública; hacer una demostración para personas que no son científicas; o una entrevista con un periodista u otros medios para una noticia o documentario científico. La participación pública también puede incluir participar en sesiones públicas con científicos y miembros del público para tener conversaciones para aprender o dar soluciones a problemas relacionados sobre la ciencia. La comunicación en línea sobre ciencia con personas que no son científicos a través de blogs, sitios web o redes sociales (por ejemplo, Facebook, Twitter/X) también es participación pública.

10. Would you say that these processes are ***effective*** ways to hear all sides of the debate about plant bioengineering? (Please check one of the following answers).

Cree que participación pública es efectiva para escuchar todos los lados del debate sobre la biotecnología en plantas?

Very effective/ **Muy efectiva**

Somewhat effective/ **Algo de efectivo**

Not too effective/ **No mucho efectivo**

Not at all effective/ **Nada efectivo**

11. Would you say that these processes are a fair way to give people a ***voice*** in decision making about plant bioengineering. (Please check one of the following answers).

Cree que participación publica es una forma justa de dar voz a las personas en la proceso de decisiones sobre biotecnología en plantas?

Very fair/ **Muy justo**

Somewhat fair/ **Algo de justo**

Somewhat unfair/ **Algo de injusto**

Very unfair/ **Muy injusto**

12. How much ***respect*** do you think that scientists leading public engagement activities about plant bioengineering have for the views of the public? (Please check one of the following answers).

Cuánto respeto cree que tienen los científicos que lideran actividades de participación pública sobre biotecnología en plantas por las opiniones del público?

A great deal of respect/ **Mucho respeto**

Some respect/ **Un poco respeto**

Not too much respect/ **No mucho respeto**

No respect at all/ **Nada respeto**

13. How would you rate the ***trustworthiness*** of scientists leading public engagement activities about plant bioengineering? (Please check one of the following answers).

Cuanto confiabilidad de los científicos que lideran actividades de participación pública sobre biotecnología en plantas?

Very trustworthy/ **Muy confiable**

Somewhat trustworthy/ **Algo de confiable**

Not too trustworthy/ **Poco confiable**

Not at all trustworthy/ **Muy poco confiable**

14. When it comes to the ***potential benefits*** of plant bioengineering, what level of benefits do you think people like you will receive? *(Please check one of the following answers).*

Qué beneficios cree que recibirán personas como usted de la biotecnología en plantas?

A very fair share of benefits/ **Una parte justa de los beneficios**

A somewhat fair share of benefits/ **Un poco parte justa de los beneficions**

A somewhat unfair share of benefits/ **Un poco parte injusta de los beneficios**

A very unfair share of benefits/ **Una parte injusta de los beneficios**

15. When it comes to the ***potential risks*** of plant bioengineering, what level of risks do you think people like you will receive? *(Please check one of the following answers).*

Qué riesgos potenciales cree que recibirán personas como usted de la biotecnología en plantas?

A very fair share of risks--**riesgos**

A somewhat fair share of risks

A somewhat unfair share of risks

A very unfair share of risks

Demographic questions (OPTIONAL)

16. Which of the following genders do you most identify with? (Please check one of the following answers).

Con cuál de los siguientes géneros se identifica más?

Male/ **Hombre**

Female/ **Mujer**

Prefer not to answer/ **Prefiero no contestar**

17. Which of the following best describes your age? (Please check one of the following answers).

Cuántos años tiene?

18-29 30-

39

40-49

50-59

60+

Prefer not to answer

18. Which of the following best describes you? (Please select **all** that apply).

Cuál de las siguientes le describe mejor?

Asian/ A**siático**

Black or African American/ A**froamericano**

Hispanic or Latino/ **Hispano o Latino**

Native American or Alaskan Native/ **Nativo americano o Nativo de Alaska**

Native Hawaiian or Pacific Islander/ **Nativo de Hawái o de las islas del Pacífico**

White/ **Blanco**

Other/ **Otro**

Prefer not to answer/ **Prefiero no contestar**

19. What is the highest level of education you have completed? (Please check one of the following answers).

Cuál es el nivel más alto de educación que ha completado?

Elementary/Primary school/ **Escuela primaria**

Middle/Secondary school/ **escuela secundaria**

High school/preparatory school diploma/ **Diploma de escuela secundaria/escuela preparatoria**

Some post-secondary education – no degree/ **Algo de educación postsecundaria, sin título**

Completed post-secondary education – certificate degree or degree/ **Educación postsecundaria, título certificado o título**

Prefer not to answer

20. What is your primary language? **Cual es su idioma principal?**

English/ **Ingles**

Spanish/ **Español**

Other: _______________

Prefer not to answer

Table 1: Survey participant demographic data

	n	%
Sex		
Male	27	46
Female	18	31
Prefer not to answer/no response	14	24
Age		
18-29	17	29
30-39	8	14
40-49	10	17
50-59	8	14
60+	8	14
Prefer not to answer/no response	8	14
Race		
Asian	4	7
Black or African American	2	3
Hispanic or Latino	34	58
Native American or Alaskan Native	1	2
White	5	8
Other	4	7
Prefer not to answer/no response	9	15
Highest Level of Education		
Elementary School	6	10
Middle School	24	41
High School	13	22
Post-Secondary, No Degree	2	3
Post-Secondary, With Degree	2	3
Prefer not to answer/no response	12	20
Preferred Language for Survey		
Spanish	59	100
